# Compact Reconfigurable Antenna with an Omnidirectional Pattern and Four Directional Patterns for Wireless Sensor Systems

**DOI:** 10.3390/s16040552

**Published:** 2016-04-16

**Authors:** Ren Wang, Bing-Zhong Wang, Wei-Ying Huang, Xiao Ding

**Affiliations:** 1 Institute of Applied Physics, University of Electronic Science and Technology of China, Chengdu 610054, China; rwang.uestc@hotmail.com (R.W.); xding@uestc.edu.cn (X.D.); 2 College of Electronic Science and Engineering, National University of Defense Technology, Changsha 410073, China; huangweiyinghwy@126.com

**Keywords:** compact antenna, omnidirectional and directional patterns, reconfigurable antenna, wireless sensor system

## Abstract

A compact reconfigurable antenna with an omnidirectional mode and four directional modes is proposed. The antenna has a main radiator and four parasitic elements printed on a dielectric substrate. By changing the status of diodes soldered on the parasitic elements, the proposed antenna can generate four directional radiation patterns and one omnidirectional radiation pattern. The main beam directions of the four directional modes are almost orthogonal and the four directional beams can jointly cover a 360° range in the horizontal plane, *i.e.*, the main radiation plane of omnidirectional mode. The whole volume of the antenna and the control network is approximately 0.70 λ × 0.53 λ × 0.02 λ, where λ is the wavelength corresponding to the center frequency. The proposed antenna has a simple structure and small dimensions under the requirement that the directional radiation patterns can jointly cover the main radiation plane of the omnidirectional mode, therefore, it can be used in smart wireless sensor systems for different application scenarios.

## 1. Introduction

In a variety of modern systems with sensors, such as temperature detection systems [1], power transmission systems [2], microwave imaging systems [3], and microwave positioning systems [4], antennas play an important role. With the development of cognitive radio in recent years [5,6], multifunctional and smart sensor networks are desired. Recently, configurable antennas, as a part of smart wireless sensor networks (WSNs), have been extensively researched [7,8,9] and widely used in mobile devices [10], cancer detection sensors [11], self-healing sensors [12], and wearable sensors [13,14]. The configurable antennas used in smart sensor systems can be divided into two kinds, frequency-reconfigurable antennas (FRA) [15,16,17,18,19] and pattern-reconfigurable antennas (PRA) [20,21,22,23,24]. FRA can be applied to telemetry [16], motion detection [17], temperature monitoring [18,19], and so on. PRA can be applied to dynamic spectrum access [21], secondary user transmission [22], direction of arrival (DoA) and received signal strength (RSS) estimation [23,24], and so on.

PRA can provide smart WSNs with several different radiation patterns, as a result, more degrees of freedom can be obtained. If the positions of the sink nodes in a WSN system are known, a directional pattern with higher gain and better spatial discrimination is a priority for a specific sink nodes [25]. There are two basic kinds of methods to switch different PRA patterns: mechanical control (MC) [26] and electrical control (EC) [27]. By contrast with MC, EC is faster and also has a smaller size for sensor nodes. In recent years, several methods with EC have been proposed to design PRA with multiple directional patterns, such as switching different main radiators [28,29,30,31], changing the status of parasitic elements [32,33,34,35], using switchable pixel layers [36,37], and using active frequency selective surfaces [38]. If the positions of the sink nodes in a WSN system are unknown, omnidirectional radiation patterns are generally required [39,40,41], especially in distributed systems or complex multipath environments. Therefore, the concept of PRA with both directional and omnidirectional patterns was proposed. To realize the concept, some design schemes have been presented.

References [42,43,44] proposed the monopole-dipole scheme (MDS) to generate both directional and omnidirectional patterns. By controlling the switches, an antenna with the MDS can act as a monopole antenna, which has an omnidirectional pattern, or a dipole antenna with a reflector, which has a directional pattern. However, the PRA with MDS can only generate one directional pattern [42,43,44] and can only be used to communicate with the sink nodes in a particular direction when directional mode is used.

Different from the MDS, the radiator array scheme (RAS) [45,46,47,48] can generate several directional patterns and an omnidirectional pattern by using different radiators. References [45,46] proposed a PRA with four Yagi radiators, which can generate an omnidirectional pattern by exciting all radiators and generate four directional patterns by exciting one of the radiators. Reference [47] presented a PRA based on a two-element dipole array model. The phase of the dipoles can be adjusted by the loaded diodes, and the antenna can generate an omnidirectional pattern and five directional patterns. The directional modes of the antennas in [45,46,47] have insufficient beamwidths in the scanning plane and cannot jointly cover the main radiation plane of the omnidirectional mode. In other word, a blindness range exists. Reference [48] introduced a PRA composed of four sector elements and each element can generate a unidirectional radiation. With a reconfigurable 1 × 4 power divider, unidirectional, bi-directional and omnidirectional radiation patterns can be obtained. The directional modes in [48] can jointly cover the main radiation plane of the omnidirectional mode and blindness range does not exist. However, this multi-element antenna in [48] has a complex structure and large dimensions because four independent antenna elements and a complex power divider network are necessary.

The PRA with multiple parasitic elements scheme (MPES) [49,50,51] may have the potential to realize a small footprint under the requirement that the directional radiation patterns can jointly cover the main radiation plane of the omnidirectional mode, because the only one main radiator of MPES may occupy a very small space and the multiple parasitic elements may generate several patterns. Based on the MPES, small dimension PRAs with two directional patterns and an omnidirectional pattern were designed in [50,51]. However, the directional patterns of these designed examples cannot jointly cover a 360° range and have a blindness area in the covering plane because of insufficient directional modes.

In this study, a compact MPES reconfigurable antenna with an omnidirectional mode and four directional modes for DoA sensor applications in the C-band is proposed. This frequency band has been chosen for its specific low absorption propagative characteristics [52,53,54]. The operation frequencies are designed in a wide band because a wide band can improve the accuracy of direction estimation. The whole dimension of the antenna and the control network is 0.70 λ × 0.53 λ × 0.02 λ, where λ is the wavelength corresponding to the center frequency. The four directional patterns can jointly cover a 360° range in the horizontal (H) plane. The proposed antenna can meet the requirement that the directional radiation patterns can jointly cover the main radiation plane of the omnidirectional mode and also has a simple structure and small dimensions. Therefore, it can be used in smart wireless sensor systems for different application scenarios, especially DoA estimation systems.

This paper is organized as follows: firstly, the geometry and operating mechanism of the reconfigurable antenna are introduced and some key parameters are analyzed. Afterwards, the simulated and measured results of the proposed antenna are demonstrated and analyzed. Finally, a summary of some reconfigurable antennas with the capacity to generate both directional and omnidirectional patterns is made.

## 2. Antenna Design and Analysis

**Figure 1:**
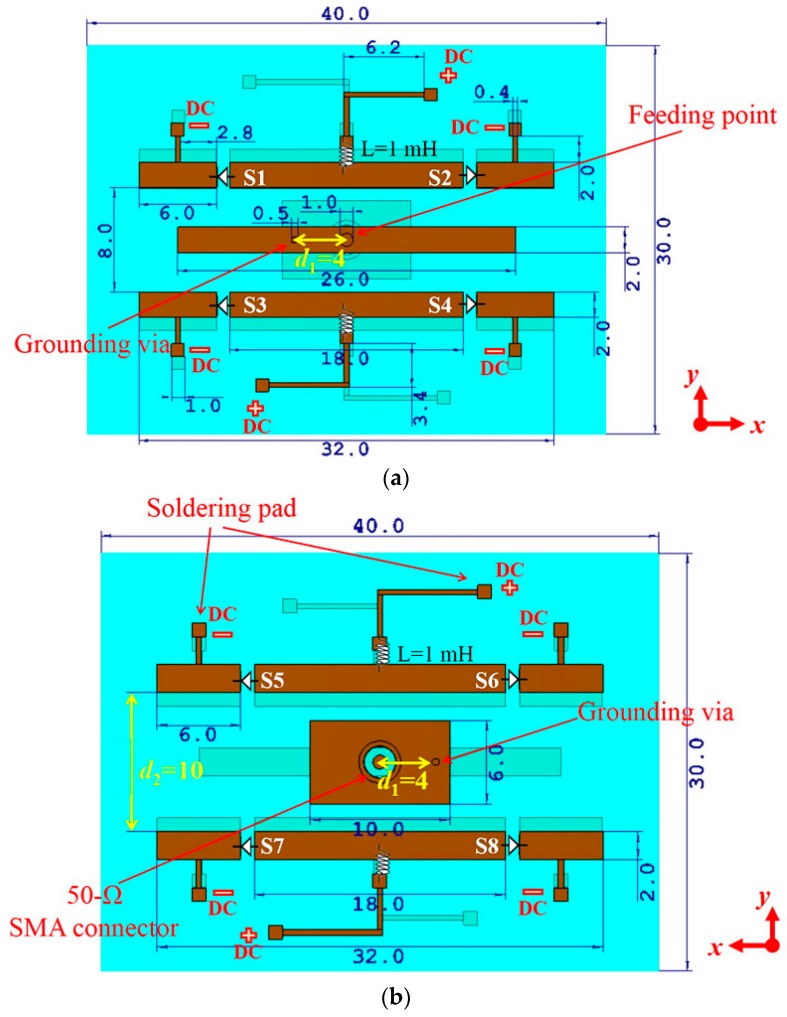
Geometry of the proposed antenna: (**a**) top view and (**b**) bottom view. The unit of the numbers is mm. S1~S8 represent PIN diodes and L represents the value of inductances. The color brown represents copper material. The dimensions are optimized by CST Microwave Studio.

The proposed antenna is composed of a dielectric substrate and two layers of copper patches printed on the substrate, as shown in Figure 1. The whole dimension of the antenna and the control network is 0.70 λ × 0.53 λ × 0.02 λ, where λ is the wavelength corresponding to the center frequency. The substrate has a thickness of 1 mm and a relative dielectric constant of 2.2.

The top side of the antenna is shown in Figure 1a. Three rectangular strips are printed on the top side. The middle strip is the main radiator fed with a 50-Ω coaxial probe and the two parasitic elements with four PIN diodes are printed on two sides of the main radiator. A feed cable has been considered attached on the SMA connector to reduce the difference between the numerical results and the experimental measurements. On each parasitic element, there are two control lines connected to the negative terminal and one control line connected to the positive terminal of the direct current (DC) control source. The line connected to the positive terminal has a length of about 0.25 λ. For the convenience of soldering, a 1 × 1 mm^2^ pad is printed at the end of each control line. In the positive terminal, inductors are soldered to isolate the RF signal. In the negative terminal, inductors are omitted because RF currents are very weak in the end of parasitic elements and the fine DC lines have a high impedance to RF signals. A similar biasing method was proposed in [32]. The back side of the antenna is shown in Figure 1b. A rectangular patch and two parasitic elements are printed on the back side. The back-side rectangle patch is connected to the top-side main radiator with a copper via and the distance *d*_1_ between the via and the feeding point has a significant influence to the impedance matching. The distance between two back-side parasitic elements is a little larger than that between two top-side parasitic elements to obtain a better impedance matching. The impacts of key parameters will be analyzed next.

To be applied for different scenarios in smart WSN systems, the proposed antenna is designed to generate four directional modes (Mode-1~Mode-4) and one omnidirectional radiation mode (Mode-5) by changing the statuses of PIN switches. In the numerical simulations, the PIN diodes are modelled as resistors and capacitors in the ON and OFF status [33,44], whose values are obtained from the corresponding data sheets. The PIN diodes used on the proposed antenna are MA4AGBLP912 diodes and they are represented by means of a capacitor of 0.02 pF in the OFF status, and a resistor of 4 Ω in the ON status, according to the MA4AGBLP912 PIN diode data sheet [55]. Table 1 shows the switch status corresponding to different reconfigurable modes. When the switches on two of the four elements are at the ON status and others are in the OFF status, a directional radiation mode is generated, while when all the switches are in the OFF status, the omnidirectional radiation mode is obtained. 

**Figure 2:**
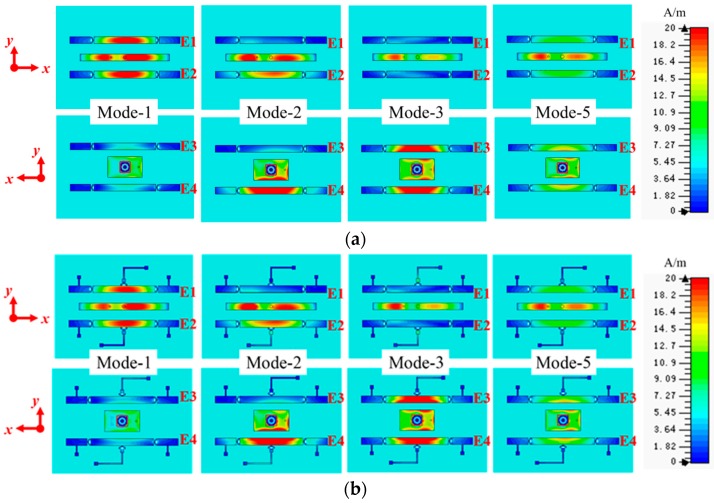
Surface current distribution at 5.5 GHz of the proposed reconfigurable antenna: (**a**) without bias network and (**b**) with bias network.

**Figure 3:**
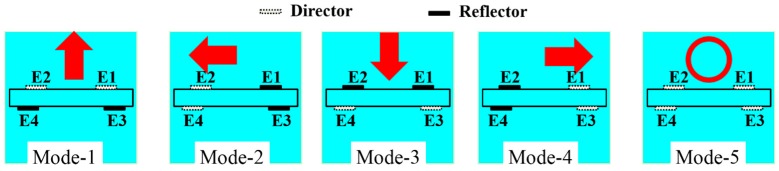
Operating mechanism of the proposed reconfigurable antenna.

Because the antenna radiation mechanism can be identified by analyzing the surface currents distributions [56], the surface current distributions at different modes of the proposed reconfigurable antenna are shown in Figure 2 to describe its radiation mechanisms. From these figures, we can see that the current distributions at a specific mode are similar whether the bias network exists or not. In different operation modes, the current intensity on each parasitic element is different. When the antenna operates at Mode-1, the currents mainly distribute on the main radiator, Element 1 (E1), and Element 2 (E2), the currents on Element 3 (E3) and Element 4 (E4) are weak. Based on the Yagi antenna mechanism [57], E1 and E2 act as directors, E3 and E4 act as reflectors in this case, therefore, the antenna will generate a directional radiation pattern and the maximum radiation is directed to the +z axis. When the antenna operates at Mode-2, the currents is mainly distributed on the main radiator, E2, and E4, the currents on E1 and E3 are weak. In this case, E2 and E4 act as directors, E1 and E3 act as reflectors, thus, the antenna will generate a directional radiation pattern and the maximum radiation is directed to the –y axis. When the antenna operates at Mode-3, the currents are mainly distributed on the main radiator, E3, and E4, the currents on E1 and E2 are weak. In this case, E3 and E4 act as directors, E1 and E2 act as reflectors, thus, the antenna will generate a directional radiation pattern and the maximum radiation is directed to the –z axis. When the antenna operates at Mode-4, the radiation direction is opposite to Mode-2 because of the structure symmetry. When the antenna operates at Mode-5, the currents are mainly distributed on the main radiator, and the currents on E1, E2, E3, and E4 are weak. In this case, the antenna radiates similar to a dipole, which has an almost omnidirectional radiation pattern. To gain some physical insight, the operating mechanisms of each radiative mode are shown in Figure 3.

**Figure 4:**
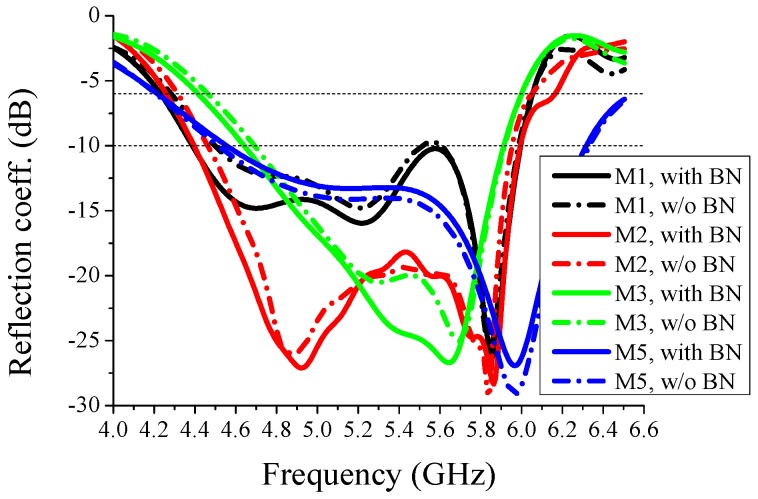
Simulated reflection coefficients of the proposed antenna with and without bias network. M1~M5 represent Mode-1~Mode-5, respectively. Two lines representing −6 dB and −10 dB are marked in the figures.

**Figure 5:**
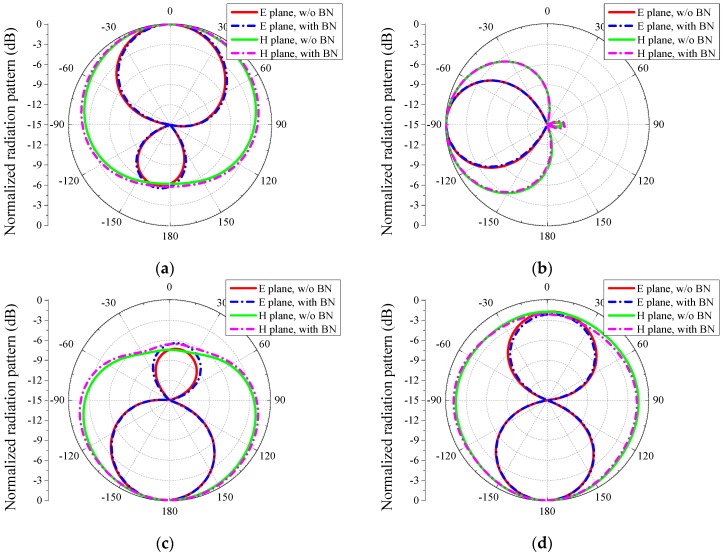
Simulated radiation patterns at 5.5 GHz of the proposed antenna with and without bias network: (**a**) Mode-1, (**b**) Mode-2, (**c**) Mode-3, and (**d**) Mode-5.

To show the effect of the bias network, reflection coefficients and radiation patterns of the proposed antenna with and without bias network are shown in Figure 4 and Figure 5, respectively. BN represents bias network in the figures. From these figures, we can see that there are few differences between the two situations, with and without bias network, both in the reflection coefficient and radiation pattern. The current distributions at a specific mode in Figure 2 are similar whether the bias network exists or not, which verifies the reasonability of Figure 4 and Figure 5. In other words, the bias network is practical and the isolation method is efficient.

Because the proposed antenna is designed to provide reconfigurable patterns in an operation band, all the modes should have the similar operation bands. In other word, the overlapped operation band of all the five modes is the operation band of the configurable antenna. Therefore, we aim to enlarge the overlapped band of the five modes by adjusting the structure parameters. As described previously, some key parameters have important effects on the impedance matching and they are analyzed below. 

**Figure 6:**
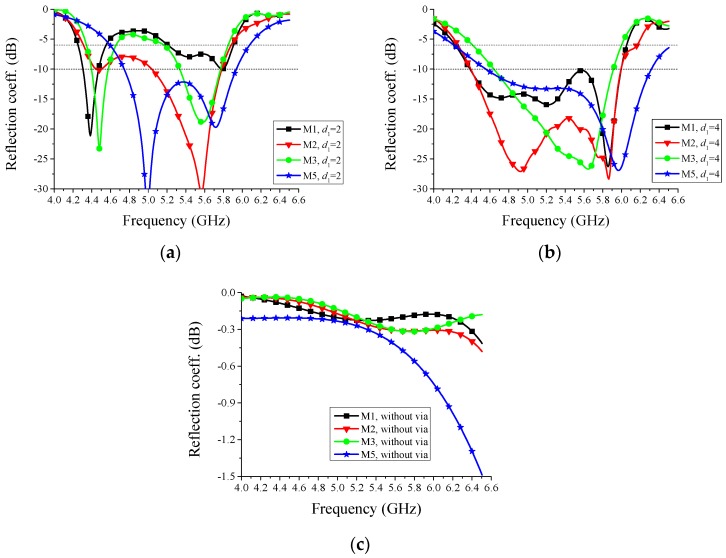
Simulated reflection coefficients corresponding to different distances *d*_1_ between feeding point and via: (**a**) *d*_1_ = 2 mm, (**b**) *d*_1_ = 4 mm, and (**c**) without via. M1~M5 represent Mode-1~Mode-5, respectively. Two lines representing −6 dB and −10 dB are marked in the figures.

The reflection coefficients corresponding to different values of *d*_1_, the distance between the feeding point and the via, are shown in Figure 6. Except the value of *d*_1_, other structure parameters all correspond to the values in Figure 1. Considering the symmetry, Mode-2 and Mode-4 may have the same simulated reflection coefficients, thus, only the results of Mode-2 are drawn in these figures. Two lines representing −6 dB and −10 dB are marked in the figures. From Figure 6, we can see that when *d*_1_ = 2 mm, the overlapped frequency band below −6 dB is from 5.25 GHz to 5.8 GHz and no overlapped band is below −10 dB. When *d*_1_ = 4 mm, the overlapped frequency band below −6 dB is from 4.4 GHz to 6.0 GHz and that below −10 dB is from 4.7 GHz to 5.9 GHz. When there is no via connecting the back-side rectangle patch and the top-side main radiator, the reflection coefficients of all the modes are very high, greater than −2 dB between 4 GHz and 6.5 GHz, which demonstrates the importance of the via. Therefore, *d*_1_ = 4 mm is chosen for the proposed antenna.

**Figure 7:**
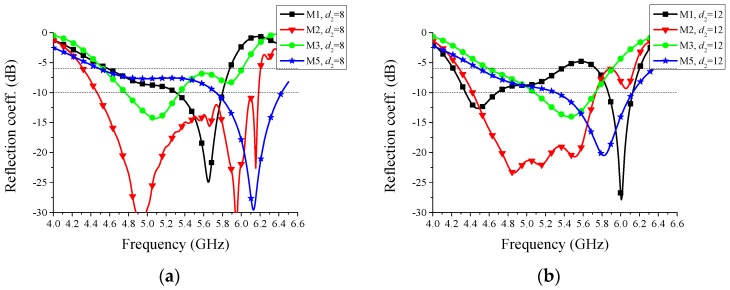
Simulated reflection coefficients corresponding to different distances *d*_2_ between the two back parasitic elements: (**a**) *d*_2_ = 8 mm and (**b**) *d*_2_ = 12 mm. M1~M5 represent Mode-1~Mode-5, respectively. Two lines representing −6 dB and −10 dB are marked in the figures.

The reflection coefficients corresponding to different values of *d*_2_, the distance between the two back parasitic elements, are shown in Figure 7. Except the value of *d*_2_, all other structure parameters, including the distance between the two top parasitic elements and *d*_1_, correspond to the values in Figure 1. In this context, we can easily know that the case of *d*_2_ = 10 mm and the case of *d*_1_ = 4 mm in Figure 6 are the same. From Figure 7, we can see that when *d*_2_ = 8 mm, the overlapped frequency band below −6 dB is from 4.6 GHz to 5.9 GHz and no overlapped band is below −10 dB. When *d*_2_ = 12 mm, the overlapped frequency band below −6 dB is from 4.6 GHz to 5.4 GHz and 5.7 GHz to 5.9 GHz, and no overlapped band is below −10 dB. The case of *d*_2_ = 10 mm is better than the cases of *d*_2_ = 8 mm and *d*_2_ = 12 mm. Therefore, *d*_2_ = 10 mm is chosen for the proposed antenna.

## 3. Results and Discussions

**Figure 8:**
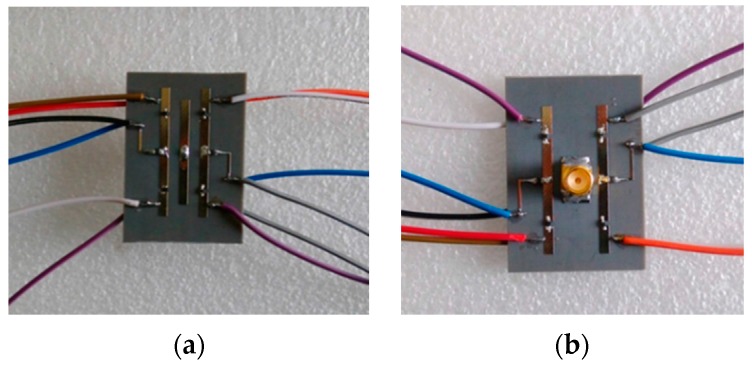
Photographs of the proposed antenna: (**a**) top view and (**b**) bottom view.

According to the structure parameters marked in Figure 1, a prototype of the proposed reconfigurable antenna was fabricated. Two photographs of the antenna are shown in Figure 8. The copper signal lines with polythene insulation layers are soldered on the soldering pads of the proposed antenna and connected to the DC control source with a voltage of 3 V to switch the PIN diodes (MA4AGBLP912, Macom Inc., Lowell, MA, USA [55]).

**Figure 9:**
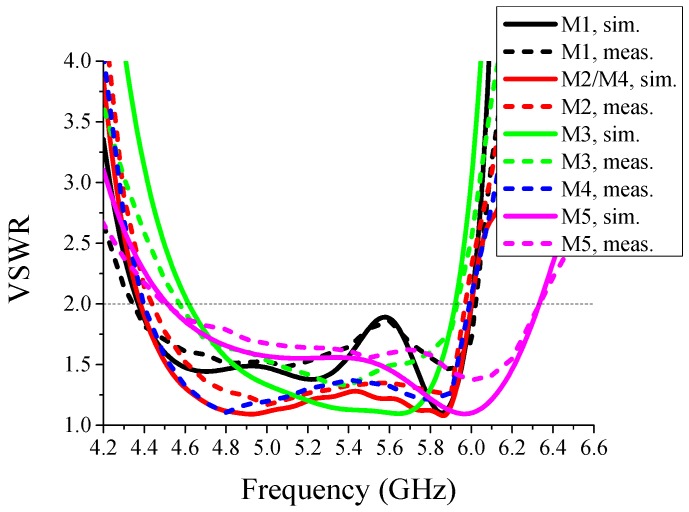
Simulated and measured VSWR of the proposed antenna. M1~M5 represent Mode-1~Mode-5, respectively. The operation bands with VSWR below 2 of all the modes include 4.6–5.9 GHz.

The simulated and measured voltage standing wave ratios (VSWR) of the proposed antenna are shown in Figure 9. From this figure, it appears that the operative band of the proposed reconfigurable antenna, *i.e.*, the overlapped frequency band, extends from 4.6 GHz up to 5.9 GHz. Moreover, a good agreement between numerical results and measurements can be observed in the figure.

**Figure 10:**
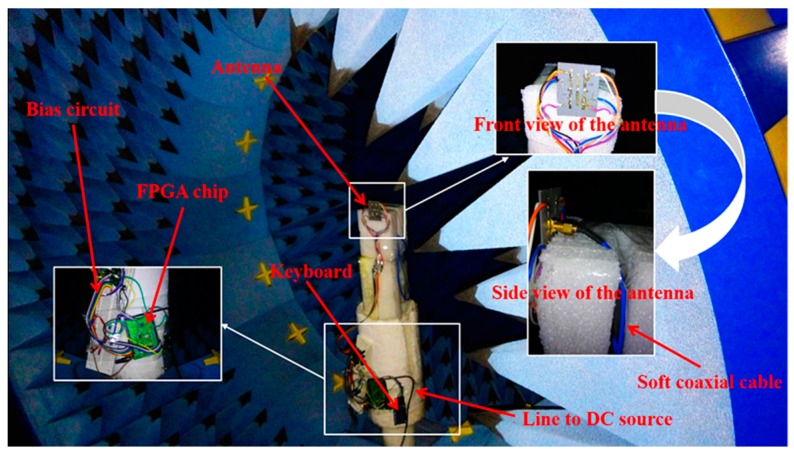
Scene layout of the antenna with SATIMO Antenna Measurement System.

The far-field patterns are measured in a microwave anechoic chamber with a SATIMO Antenna Measurement System, as shown in Figure 10. Regarding the pattern of Mode-3, it radiates towards the -z direction, where a coaxial cable exists to feed the antenna. Even though the feeding cable has been modeled on the practically SMA connector in the simulation, the coaxial cable connected to the measurement equipment may have serious effects on the radiation pattern if the antenna is installed vertically to the whole cable. To reduce the effect of the cable, the antenna is connected to a soft coaxial cable and the soft cable is turned to be parallel to the antenna when it extends away. Therefore, the most of the cable are along the null radiation directions of the antenna and the part near the antenna has been considered in the simulation. As a result, the effect of feeding cable can be reduced. In addition, the DC control lines are turned to be parallel to the antenna when it extends away, similarly to the feeding cable, to reduce the effect. The control system, including DC lines, FPGA chip (Core EP4CE6E22C8N, Waveshare Inc., Lowell, MA, USA), FPGA bias circuit, and keyboard, is arranged away from the antenna and along the null radiation directions. 

**Figure 11:**
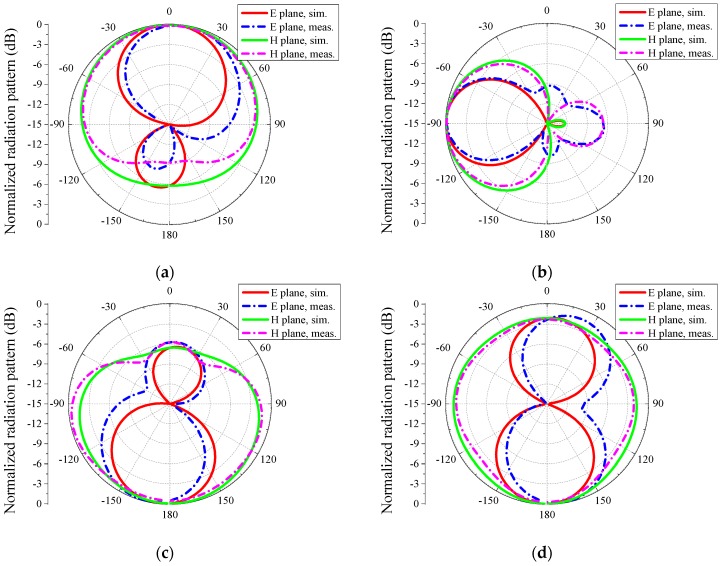
Simulated and measured far-field radiation patterns at 5.5 GHz of the proposed antenna: (**a**) Mode-1, (**b**) Mode-2, (**c**) Mode-3, and (**d**) Mode-5. The H-plane patterns of Modes1~Mode-3 are directional and that of Mode-5 is omnidirectional.

The simulated and measured radiation patterns of the proposed antenna corresponding to each mode are shown in Figure 11. Considering the symmetry, patterns of Mode-4 are not drawn in these figures. Detailed information on the radiation patterns corresponding to Figure 11 is shown in Table 2.

From Figure 11, in the case of Mode-1, the simulated and measured main beams in the H plane of the proposed antenna direct to θ = 0° and θ = 1°, respectively. In the case of Mode-2, the simulated and measured main beams in the H plane both direct to θ = −90°. In the case of Mode-3, the simulated and measured main beams in the H plane direct to θ = −180° and θ = −179°, respectively. The directions of the directional modes are almost orthogonal. The simulated and measured half-power beamwidths (HPBW) in the H plane of the directional modes are from 110° and 220° and from 107° and 217°, respectively. Therefore, the four directional radiation modes can jointly cover a 360° range in the H plane. When the proposed antenna operates at Mode-5, the simulated and measured HPBW in the H plane are both 360° and omnidirectional radiations are obtained. Thus, by switching the PIN diodes, the proposed reconfigurable antenna can generate an omnidirectional pattern and four directional patterns and the directional radiation patterns can jointly cover the main radiation plane of the omnidirectional mode. 

In addition, the simulated and measured patterns have evident differences, especially in the E plane. In Figure 11a,c,d an obvious pattern-tilting phenomenon occurs in the E plane of the measured patterns. In Figure 11b, large back lobes appear in both the E plane and H plane of the measured patterns, compared with the simulated patterns. The pattern-tilting and back lobe phenomena may result from the DC control wires. Fortunately, the effect of wires are not too serious because of our measurement setups in Figure 10.

**Figure 12:**
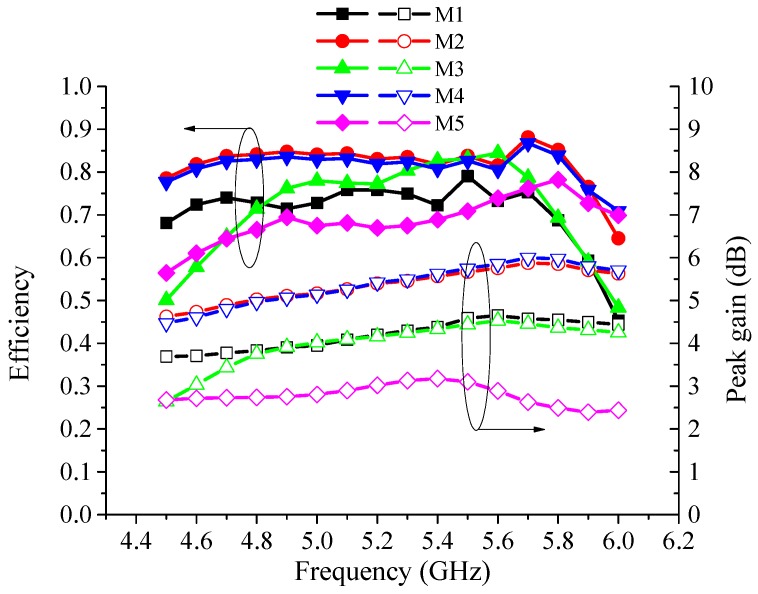
Measured efficiencies and peak gains of the proposed antenna corresponding to different modes.

The measured efficiencies and peak gains corresponding to different radiation modes are shown in Figure 12. When the proposed reconfigurable antenna operates at all five modes, the measured efficiencies are more than 60% in the 4.6 GHz-5.9 GHz operation frequency band. The peak gains at Modes-2/4, Modes-1/3, and Mode-5 are from 5 dB to 6 dB, from 3.5 dB to 4.5 dB, and from 2.5 dB to 3 dB, respectively, in the operation band. It is notable that Mode-1 has a lower gain compared to Mode-2, even though they are both directional patterns. From Figure 11 and Table 2, we can see that the beamwidths of Mode-1 are much wider than those of Mode-2, especially in the H plane, which results to a lower gain of Mode-1. Considering the geometry in Figure 1 and the operating mechanism in Figure 3, the radiation mechanism of Mode-2 is very similar to the Yagi antenna, a typical directional antenna. When the antenna operates at Mode-1, the distance between directors and reflectors is very small, thus, the directionality will be weakened and the gain will decline [57].

As mentioned in the Introduction, three basic methods to design reconfigurable antennas with both directional and omnidirectional patterns have been proposed. To compare the detailed characteristics, some design examples are summarized in Table 3. The impedance bandwidth section is calculated with the overlapped frequency band of all the modes of the corresponding antenna. The blindness range section is to display whether the directional radiation patterns of the antenna can jointly cover a particular plane, *i.e.*, main radiation plane of the omnidirectional mode. The peak gain section shows the maximum gain among all the modes of the corresponding antenna. The beamwidth section shows the minimum (before “/”) and maximum (after “/”) beamwidths of the directional radiation patterns in the scanning plane, and the symbol “~” represents that the beamwidth value is estimated from the patterns in the corresponding paper which does not show the beamwidth. The volume section with the unit of λ^3^ is the product of length, width, and height, where λ is the wavelength corresponding to the center operation frequency. The area section with the unit of λ^2^ is the product of length and width. The structure complexity section depends on the available processing method. If an antenna needs to be assembled with several printed circuit boards or metal plates, it is considered complex.

References [42,44] based on the MDS method only have one directional mode, thus, the directional mode cannot jointly cover a radiation plane. References [46,47] based on RAS method have four directional radiation modes in the main radiation plane of omnidirectional mode, but the beamwidths in the scanning plane of all the four directional mode are less than 90°, therefore, blindness range exists. References [50,51] based on the MPES method have two directional radiation modes in the main radiation plane of omnidirectional mode, the beamwidths in the scanning plane of all the directional mode are more than 90° but less than 180° and blindness range exists. Reference [48] can generate multiple modes by combining four antenna elements and a feeding network. Its directional modes can jointly cover the main radiation plane of the omnidirectional mode and blindness range does not exist. However, its dimension is large because of the multi-antenna and feeding network. As a comparison, the proposed antenna in this paper has a small dimension under the requirement that the directional radiation patterns can jointly cover the main radiation plane of the omnidirectional mode. In addition, the antennas in [48,50,51] and the proposed antenna have a relatively wide band; the antennas in [42,44,48,50] and the proposed antenna have a relatively high gain; the antennas in [42,46,51] and the proposed antenna have a simple structure. In a word, the two goals of the proposed work, small dimension and no blindness range, are obtained and other important parameters of the proposed antenna is not bad as a pattern reconfigurable antenna.

## 4. Conclusions

In this paper, a compact reconfigurable antenna with an omnidirectional mode and four directional modes is proposed. The patterns of the four directional modes can jointly cover the main radiation plane of the omnidirectional mode, which means that the main goal of the proposed work is achieved. Compared to the radiator array scheme that can also achieve this goal, the proposed method with multiple parasitic elements needs only one radiator and no power divider networks, thus, the dimensions can be small. However, the messy DC control wires may affect the radiation pattern. In practical applications, the DC control wires should be integrated with the antenna according to the given situation. The proposed antenna can be used in smart wireless sensor systems of different application scenarios, especially DoA estimation systems in the C-band.

## Figures and Tables

**Table 1:** Switch statuses and the corresponding reconfigurable modes.

	Mode-1	Mode-2	Mode-3	Mode-4	Mode-5
S1&S2	OFF	ON	ON	OFF	OFF
S3&S4	OFF	OFF	ON	ON	OFF
S5&S6	ON	ON	OFF	OFF	OFF
S7&S8	ON	OFF	OFF	ON	OFF

**Table 2:** Detailed information about the radiation patterns corresponding to Figure 11.

		Main Beam Direction		Half-Power Beamwidth		Front-to-Back Ratio	
		E plane	H plane	E plane	H plane	E plane	H plane
Mode-1	Sim.	8°	0°	84°	220°	5.5 dB	6 dB
	Meas.	15°	1°	90°	192°	8 dB	9 dB
Mode-2	Sim.	−90°	−90°	61°	110°	13 dB	12 dB
	Meas.	−89°	−90°	59°	107°	7 dB	7 dB
Mode-3	Sim.	−187	−180°	75°	215°	6.5 dB	6.5 dB
	Meas.	−190°	−179°	80°	217°	5.5 dB	5.5 dB
Mode-5	Sim.	–	–	–	360°	–	–
	Meas.	–	–	–	360°	–	–

**Table 3:** Summary of some reconfigurable antennas with the capacity to generate both directional and omnidirectional patterns.

Reference	Basic Method	Impedance Bandwidth	Blindness Range	Peak Gain	Beamwidth	Volume (λ^3^)	Area (λ^2^)	Structure Complexity
[42]	MDS	6.5%	Yes	5.3 dB	~180°	0.002	0.24	No
[44]	MDS	3.7%	Yes	6.6 dB	~90°	0.009	0.54	Yes
[46]	RAS	8.2%	Yes	3.6 dB	65°/65°	0.006	0.45	No
[47]	RAS	6.6%	Yes	4 dB	59°/65°	0.007	0.08	Yes
[48]	RAS	19%	No	7.1 dB	140°/140°	3.648	2.16	Yes
[50]	MPES	16%	Yes	6.4 dB	~120°	0.259	0.39	Yes
[51]	MPES	127%	Yes	2.1 dB	~150°	0.015	0.45	No
Proposed	MPES	24%	No	5.9 dB	107°/217°	0.007	0.37	No

## References

[1] Sun J., Zhao K., Jiang T. (2014). A multipoint correction method for environmental temperature changes in airborne double-antenna microwave radiometers. Sensors.

[2] Cao H., Jiang F., Liu J., Cai W., Tang M., Tan X. (2015). A CSRR-fed SIW cavity-backed fractal patch antenna for wireless energy harvesting and communication. Sensors.

[3] Islam M.T., Islam M.M., Samsuzzaman M., Rashed M., Faruque I., Misran N. (2015). A negative index metamaterial-inspired UWB antenna with an integration of complementary SRR and CLS unit cells for microwave imaging sensor applications. Sensors.

[4] Fujii K., Sakamoto Y., Wang W., Arie H., Schmitz A., Sugano S. (2015). Hyperbolic positioning with antenna arrays and multi-channel pseudolite for indoor localization. Sensors.

[5] Lin S.C., Chen K.C. (2014). Improving spectrum efficiency via in-network computations in cognitive radio sensor networks. IEEE Trans. Wirel. Commun..

[6] Zubair S., Fisal N. (2014). Reliable geographical forwarding in cognitive radio sensor networks using virtual clusters. Sensors.

[7] Costantine J., Tawk Y., Barbin S.E., Christodoulou C.G. (2015). Reconfigurable antennas: design and applications. IEEE Proc..

[8] Christodoulou C.G., Tawk Y., Lane S.A., Erwin S.R. (2012). Reconfigurable antennas for wireless and space applications. IEEE Proc..

[9] Hussain R., Sharawi M.S. (2014). A cognitive radio reconfigurable MIMO and sensing antenna system. IEEE Antennas Wirel. Propag. Lett..

[10] Lin Y.C., Yu C.Y., Li C.M., Liu C.H., Chen J.P., Chu T.H. (2014). An ionic-polymer-metallic composite actuator for reconfigurable antennas in mobile devices. Sensors.

[11] Caratelli D., Massaro A., Cingolani R., Yarovoy A.G. (2012). Accurate time-domain modeling of reconfigurable antenna sensors for non-invasive melanoma skin cancer detection. IEEE Sens. J..

[12] Yuan S., Qiu L., Gao S., Tong Y., Yang W. (2012). Providing self-healing ability for wireless sensor node by using reconfigurable hardware. Sensors.

[13] Burnett D.C., Smarr B.L., Mesri S.M., Kriegsfeld L.J., Pister K.S. Reconfigurable, wearable sensors to enable long-duration circadian biomedical studies. Proceedings of the 9th International Conference on Body Area Networks.

[14] Chen C.A., Chen S.L., Huang H.Y., Luo C.H. (2012). An efficient micro control unit with a reconfigurable filter design for wireless body sensor networks (WBSNs). Sensors.

[15] Erfani E., Nourinia J., Ghobadi C., Niroo-Jazi M., Denidni T.A. (2012). Design and implementation of an integrated UWB/reconfigurable-slot antenna for cognitive radio applications. IEEE Antennas Wirel. Propag. Lett..

[16] Kim Y.R., Woo J.M. (2012). Electrically tunable small microstrip antenna using interdigital plate loading for telemetry sensor applications. Electron. Lett..

[17] Costantine J., Tawk Y., Christodoulou C.G. (2013). Motion-activated reconfigurable and cognitive radio antenna systems. IEEE Antennas Wirel. Propag. Lett..

[18] Qiao Q., Zhang L., Yang F., Yue Z., Elsherbeni A.Z. (2013). Reconfigurable sensing antenna with novel HDPE-BST material for temperature monitoring. IEEE Antennas Wirel. Propag. Lett..

[19] Yang F., Qiao Q., Virtanen J., Elsherbeni A.Z., Ukkonen L., Sydanheimo L. (2012). Reconfigurable sensing antenna: a slotted patch design with temperature sensation. IEEE Antennas Wirel. Propag. Lett..

[20] Catarinucci L., Guglielmi S., Colella R., Tarricone L. (2014). Pattern-reconfigurable antennas and smart wake-up circuits to decrease power consumption in wsn nodes. IEEE Sens. J..

[21] Wanuga K., Gulati N., Saarnisaari H., Dandekar K.R. Online learning for spectrum sensing and reconfigurable antenna control. Proceedings of 2014 9th International Conference on Cognitive Radio Oriented Wireless Networks and Communications.

[22] Alaa A., Ismail M., Tawfik M. (2016). Random aerial beamforming for underlay cognitive radio with exposed secondary users. IEEE Trans. Veh. Technol..

[23] Hakkarainen A., Werner J., Gulati N., Patron D. Reconfigurable antenna based DOA estimation and localization in cognitive radios: Low complexity algorithms and practical measurements. Proceedings of the 2014 9th International Conference on Cognitive Radio Oriented Wireless Networks and Communications.

[24] Werner J., Wang J., Hakkarainen A., Cabric D. (2016). Performance and cramer-rao bounds for DOA/RSS estimation and transmitter localization using sectorized antennas. IEEE Trans. Veh. Technol..

[25] Guzmán-Quirós R., Martínez-Sala A., Gómez-Tornero J.L., García-Haro J. (2016). Integration of directional antennas in an RSS fingerprinting-based indoor localization system. Sensors.

[26] Tawk Y., Costantine J., Avery K., Christodoulou C.G. (2011). Implementation of a cognitive radio front-end using rotatable controlled reconfigurable antennas. IEEE Trans. Antennas Propag..

[27] Qin P.Y., Guo Y.J., Weily A.R., Liang C.H. (2012). A pattern reconfigurable U-slot antenna and its applications in MIMO systems. IEEE Trans. Antennas Propag..

[28] Sarrazin J., Mahé Y., Avrillon S., Toutain S. (2009). Pattern reconfigurable cubic antenna. IEEE Trans. Antennas Propag..

[29] Cai X., Wang A.G., Ma N., Leng W. (2012). A novel planar parasitic array antenna with reconfigurable azimuth pattern. IEEE Antennas Wirel. Propag. Lett..

[30] Lai M.I., Wu T.Y., Hsieh J.C., Wang C.H. (2008). Compact switched-beam antenna employing a four-element slot antenna array for digital home applications. IEEE Trans. Antennas Propag..

[31] Eom S.H., Seo Y., Lim S. (2015). Pattern switchable antenna system using inkjet-printed directional bow-tie for bi-direction sensing applications. Sensors.

[32] Ding X., Wang B.Z. (2013). A novel wideband antenna with reconfigurable broadside and endfire patterns. IEEE Antennas Wirel. Propag. Lett..

[33] Li M., Xiao S.Q., Wang Z., Wang B.Z. (2014). Compact surface-wave assisted beam-steerable antenna based on HIS. IEEE Trans. Antennas Propag..

[34] Qin P.Y., Guo Y.J., Ding C. (2013). A beam switching quasi-yagi dipole antenna. IEEE Trans. Antennas Propag..

[35] Gu C., Gao S., Liu H., Luo Q. (2015). Compact smart antenna with electronic beam-switching and reconfigurable polarizations. IEEE Trans. Antennas Propag..

[36] Rodrigo D., Cetiner B.A., Jofre L. (2014). Frequency, radiation pattern and polarization reconfigurable antenna using a parasitic pixel layer. IEEE Trans. Antennas Propag..

[37] Ji L.Y., Guo Y.J., Qin P.Y., Gong S.X. (2015). A reconfigurable partially reflective surface (PRS) antenna for beam steering. IEEE Trans. Antennas Propag..

[38] Zhang L., Wu Q., Denidni T.A. (2013). Electronically radiation pattern steerable antennas using active frequency selective surfaces. IEEE Trans. Antennas Propag..

[39] D'Hoe K., Ottoy G., Goemaere J.P., Strycker L. (2008). Indoor room location estimation. Adv. Electr. Comput. Eng..

[40] Chiu Y.M., Wang K., Jan R.H., Hu Y.J., Ku T.H. An efficient room-based indoor localization scheme for wireless sensor networks. Proceedings of the 5th Workshop on Wireless Ad Hoc and Sensor Networks.

[41] Lo C.-C., Chen C.-C., Tseng Y.-C., Chiang J.-C., Feng K.-C., Kuo L.-C., Wang Y.-C. A room-based localization system using wireless triggers and pattern matching techniques. Proceedings of the IEEE VTS Asia Pacific Wireless Communications Symposium.

[42] Kang W.S., Park J.A., Yoon Y.J. (2008). Simple reconfigurable antenna with radiation pattern. Electron. Lett..

[43] Hwang K.S., Ahn J., Kim K.J., Yoon H.K., Yoon Y.J. Pattern reconfigurable antenna for a wireless sensor network sink node. Proceedings of the 2010 Asia-Pacific Microwave Conference Proceedings.

[44] Kim K., Hwang K., Ahn J., Yoon Y. (2012). Pattern reconfigurable antenna for wireless sensor network system. Electron. Lett..

[45] Patron D., Piazza D., Dandekar K.R. (2013). Wideband planar antenna with reconfigurable omnidirectional and directional radiation patterns. Electron. Lett..

[46] Patron D., Daryoush A.S., Dandekar K.R. (2014). Optical control of reconfigurable antennas and application to a novel pattern-reconfigurable planar design. IEEE J. Lightw. Technol..

[47] Bai Y.Y., Xiao S., Liu C., Shuai X., Wang B.Z. (2013). Design of pattern reconfigurable antennas based on a two—element dipole array model. IEEE Trans. Antennas Propag..

[48] Row J.S., Tsai C.W. (2016). Pattern reconfigurable antenna array with circular polarization. IEEE Trans. Antennas Propag..

[49] Tawk Y., Christodoulou C.G., Costantine J., Barbin S.E. A frequency and radiation pattern reconfigurable antenna system with sensing capabilities for cognitive radio. Proceedings of the 2012 IEEE Antennas and Propagation Society International Symposium.

[50] Ren J., Yang X., Yin J., Yin Y. (2015). A novel antenna with reconfigurable patterns using H-shaped structures. IEEE Antennas Wirel. Propag. Lett..

[51] Aboufoul T., Parini C., Chen X., Alomainy A. (2013). Pattern-reconfigurable planar circular ultra-wideband monopole antenna. IEEE Trans. Antennas Propag..

[52] Partington K.C., Flach J.D., Barber D., Isleifson D., Meadows P.J., Verlaan P. (2010). Dual-polarization C-band radar observations of sea ice in the amundsen gulf. IEEE Trans. Geosci. Remote Sens..

[53] Jeon S.S., Miyamoto R.Y., Wang Y., Qian Y., Itoh T. Direction-of-arrival estimation using a single-card C-band receiver array. Proceedings of the 2000 Asia-Pacific Microwave Conference.

[54] Scarchilli G., Goroucci E., Chandrasekar V., Seliga T.A. (1993). Rainfall estimation using polarimetric techniques at c-band frequencies. J. Appl. Meteorol..

[55] MACOM/PIN Switch and Attenuator Diodes/AlGaA PIN Diode/MA4AGBLP912. https://www.macom.com/products/product-detail?partNumber=MA4AGBLP912.

[56] Caratelli D., Cicchetti R., Bit-Babik G., Faraone A. (2007). Circuit model and near-field behavior of a novel patch antenna for wwlan applications. Microw. Opt. Technol. Lett..

[57] Balanis C.A. (1997). Antenna Theory, Analysis and Design.

